# Dissecting transmission to understand parasite evolution

**DOI:** 10.1371/journal.ppat.1012964

**Published:** 2025-03-25

**Authors:** Luís M. Silva, Kayla C. King, Jacob C. Koella

**Affiliations:** 1 Institute of Biology, University of Neuchâtel, Neuchâtel, Switzerland; 2 Department of Zoology, University of British Columbia, University Boulevard, Vancouver, British Columbia, Canada; 3 Department of Microbiology & Immunology, University of British Columbia, Health Sciences Mall, Vancouver, British Columbia, Canada; 4 Department of Biology, University of Oxford, Oxford, United Kingdom; University of Mississippi Medical Center, UNITED STATES OF AMERICA

## Abstract

Parasite transmission is a complex, multi-stage process that significantly impacts host–parasite dynamics. Transmission plays a key role in epidemiology and virulence evolution, where it is expected to trade off with virulence. However, the extent to which classical models on virulence–transmission relationships apply in the real world is unclear. This insight piece proposes a novel framework that breaks transmission into three distinct stages: within-host infectiousness, an intermediate between-host stage (biotic or abiotic), and new host infection. Each stage is influenced by intrinsic and extrinsic factors to the parasite, which together will determine its transmission success. Analyzing the transmission stages separately and how they affect each other might enhance our understanding of which host-, parasite- or environmental-driven factors might shape parasite evolution and inform us about new effectors to act on when designing disease control strategies.

## 1. Introduction

**Fig 1:**
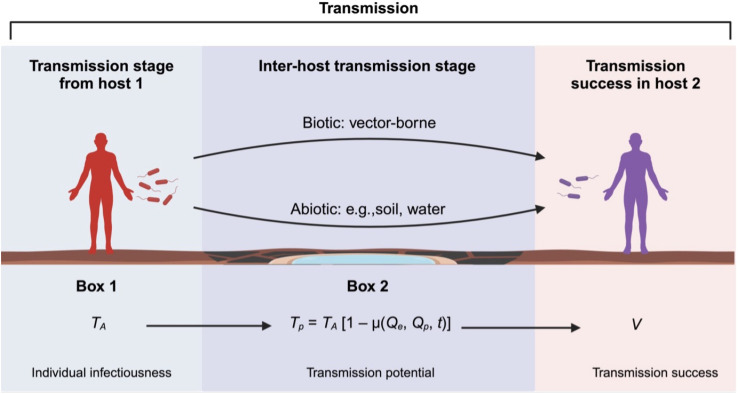
Stages of parasite transmission. Illustration of the different stages for a parasite to successfully transmit into a new host. The production rate of infective cells in host 1 (*T*_***A***_) [17,40] will impact its transmission potential (*T*_*p*_) after a biotic or abiotic stage outside the main host, which is affected by several intrinsic and extrinsic parasite factors. *Tp* will impact the chances of infection success in a new host, reflecting the full parasite fitness or transmission (V)—figure produced in Biorender.com.

Parasites are fundamentally driven to maximize their reproductive success, i.e., transmission rate to new hosts. This goal drives investment in machinery/traits that maximize transmission rate and ensure the establishment of successful infections in new hosts. Transmission rate and success are then key indicators of parasite fitness [1,2]. They can be defined as the number of secondary hosts infected by a host within a given time. It reflects the parasite’s ability to infect a host, to survive and reproduce within it, and then to infect a new host. Several factors can influence and maintain variability in this transmission process, such as the nutritional or dietary status during the development of both host and parasite [3–7]. A poor nutritional status affects the host–parasite interaction, as host immunity might be constrained, and parasite replication slowed down due to competition for resources [8–12]. Parasite transmission is evidently a complex, multi-stage process within and among hosts (Fig 1). The extent to which a parasite invests in each transmission stage may vary depending on host conditions, parasite life history, or environment. Constraints at any one stage can significantly impact the overall transmission process and, consequently, parasite fitness.

Research on parasite transmission is vital for understanding and predicting its evolution, which has major consequences for epidemiology and virulence (i.e., detrimental effects of infection on its host [13]). In recent years, epidemiological studies have integrated transmission heterogeneity into forecasts of parasite evolutionary trajectories. Superspreading, for example, is when a small number of infected individuals cause a disproportionately large number of new infections [7,14–16]. This phenomenon can undermine control measures and contribute to ongoing epidemics, leading to more frequent disease outbreaks [17,18]. Research on transmission also plays a vital role in the evolution of virulence, where the two traits are expected to be linked. Most major hypotheses, disease control strategies, and predictions regarding virulence evolution [19] are based mainly on the prevailing theory of virulence evolution [2,20,21] due to its easy and broad application. This theory postulates a trade-off between a parasite’s transmission rate and its infection virulence [20], meaning a parasite that evolves to kill the host too quickly may not get the chance to be transmitted. This theory has been crucial to estimating and tackling parasite evolution that might jeopardize the survival of populations and species with low genetic diversity (e.g., cattle, endangered species) and, therefore, more susceptible to novel infections [22,23]. Since its introduction approximately 50 years ago, this trade-off theory has found empirical and theoretical support [19,21,24–27]. There are nonetheless questions about its generality across host-parasite systems, with several studies not observing the trade-off or finding that it does not apply to types of infection (e.g., tissue tropism) or transmission modes (e.g., obligate killer parasites) [27–34].

The transmission rate in standard SIR models is often represented by a single parameter: the basic reproductive number (*R*_*0*_). This parameter is defined as *“the average number of secondary infections caused by a single, infected individual in a completely susceptible population”* [20,35]. *R*_*0*_ is a valuable tool for predicting whether an infectious disease will become an epidemic [36,37]. It does not, however, account for the variability in transmission rate among individuals [17] or the intricate interactions of intrinsic and extrinsic parameters that influence the transmission process and its outcome [3,38]. To better understand the impact of host heterogeneity in transmission, Lloyd-Smith and colleagues (2005) introduced the concept of *“individual reproduction number”* (or *V*). This metric represents the expected number of secondary cases caused by each infected individual [17]. By focusing on individual contributions rather than the population average, this concept accounts for variability in transmission among individuals, which can lead to different epidemiological predictions and necessitate more targeted disease control measures [18,39]. VanderWaal and Ezenwa (2016) expanded this transmission framework to include key aspects of infection and host–parasite interactions that are likely to impact *V*, such as infectiousness, contact rate, and the length of the infectious period [40]. While these [17,40] and other refinements [41] represent a significant advancement by addressing host heterogeneity and its effects, it still overlooks other important factors contributing to the complexity of transmission rate variability [21]. These factors include differences in host contact/behavior [39,42,43], immunocompetence [44–46], host- and parasite-specific factors like parasite load and symptom severity [38,44], and environmental factors such as population density [33,47,48]. Additionally, other factors, such as the protective role of the microbiome [49] or age [50], also play a role in influencing a host’s infectiousness and parasite reproductive number.

In this article, we address why and how existing frameworks should include the environment outside of the host, and we tackle the ambiguity regarding the metrics of different transmission stages. Note that although most parasites have an environment outside their primary host (abiotic or biotic), some do not. That is the case for several sexually transmitted infections, such as the human immunodeficiency virus (HIV), which often skips this stage and is directly transmitted from one host to the other. This framework does not apply to those. As stated by McCallum and colleagues (2017), a single transmission term hinders us from understanding the dynamics of transmission but also the relationship between different transmission stages and host-parasite traits. In their study, they showed with theoretical modeling how decomposing transmission can highlight nonlinear relationships between various components of transmission [41] Hence, to enhance our understanding of the relationship between the transmission process and parasite evolution, we enhanced McCallum’s deconstruction of the transmission process and proposed an advanced framework that not only breaks down the transmission process into distinct stages but also highlights and formalizes the different factors impacting each stage for empirical testing. Each stage is open to its own set of factors that might influence stage-specific transmission rate metrics or *V*. This framework is designed to be simple enough for broad application across various infection types, yet flexible enough to accommodate different aspects of the parasite’s transmission cycle, whether intrinsic or extrinsic. Moreover, we also defined the following transmission stages and respective metrics: (1) initial primary host and infectiousness, i.e., parasite numbers released to the next stage; (2) time between primary hosts and transmission potential, i.e., number of parasites that survive the time (t) outside the host; (3) infection of a new primary host and transmission success, i.e., the parasite can successfully establish an infection in the secondary host (Fig 1). We believe that by formally decomposing the transmission process into its stages, each with its respective metric, we might acquire insights into parasite evolution, the limitations to its evolvability and which factors are responsible for it.

## 2. Transmissibility and infectiousness

Before transmission to a new host/environment, a parasite must navigate its development within its primary host and address potential constraints the host imposes. These constraints can arise from the host immune strategy [5,51,52] to the resources available for the parasite to sequester and then utilize [53–55], which can also be affected by host microbiota [49,56]. For instance, there is contrasting evidence that microbiota can mediate protection against a parasite but also favor the evolution of virulence in certain conditions [49,57,58]. Nevertheless, a parasite can still manipulate the host’s behavior [59] and physiology [60–62] to enhance its chances of transmission. Among the factors influencing this stage, two are particularly relevant: the parasite load and the duration of the infection [40].

A striking example of how within-host factors can influence parasite dynamics and evolution is through the defense strategy employed. Hosts may opt to resist or tolerate a parasite [49,55,63,64]. Resistance involves limiting the number of parasitic cells, while tolerance reduces the damage caused by the infection without directly affecting parasite growth [55]. Tolerance allows a higher parasite load to accumulate within the host. As an example of this in healthcare, a vaccine, such as the one against the common flu, would induce a higher immune response and, therefore, act through resistance instead of tolerance.

Nevertheless, tolerance vaccines have been in the making for a few years now, aiming to decrease the cost of the infection instead of killing the parasite [65–67]. Parasite load within a host is evidently linked to its infectiousness, and it is fair to expect superspreading to evolve in these circumstances. At its core, superspreading is seen when infected hosts can transmit higher parasite loads with fewer visible symptoms or costs than others [15,16]. This phenomenon might entail a population-wide heterogeneity in transmission, and the lack of symptoms in these individuals might lead to weak disease surveillance. Indeed, this variation has been observed in infections such as SARS-CoV-2 [7,68], MERS-CoV [69], Q fever [70], and tuberculosis [71], to name a few. Given the nature of tolerance, it is fair to assume this strategy might lead to more contagious infections than resistance [72], although there is no empirical evidence for it yet. Differences in how hosts allocate resources or invest into resistance or tolerance [73–75] will result in a mix of highly contagious superspreader hosts and individuals who contribute minimally to the populational transmission rate.

Transmissibility, as the ability to transmit a given infection, is determined not only by the number of parasite cells produced during a certain infection period but also by their quality and infectious potential. These factors, in turn, can be grouped into physiological or behavioral mechanisms [17,40] which may evolve independently or together. Physiological mechanisms involve factors affecting the length of the infectious period (*I*_*P*_) and the infectiousness of the parasites produced (*β*_*p*_). Behavioral mechanisms include host social aspects, such as population density or increased contact rates (*β*_*c*_), which are dependent on host motility and can be genetically governed [76]. For instance, the transmission of the parasite *Plasmodium falciparum* is associated with its density during its infectious stage, which is regulated physiologically by the host immune system [60]. Nonetheless, the contagious stage also increases the human attractiveness to mosquitoes and behaviorally increases the chances of transmission [60] (so, its infectiousness). Consequently, both types of mechanisms can differently affect parasite reproductive numbers through variation in some of the main components of transmission: the number and quality of parasites within their host. Measured on an appropriate scale, these can be multiplied to give the ability of transmission (*T*_*A*_).


$$T_{A}=\beta_{p}\times \beta_{c}\times I_{P}$$

Numerous environmental and genetic factors affect each of these parameters, such as the host’s nutritional status [5,51,52] and immunocompetence [44–46] and the parasite’s reproductive rate in optimal conditions. Moreover, such factors may depend on each other. For example, hosts with a high parasite load may have a lower contact rate or a shorter infectious period. It is important to note that *β*_*c*_ represents behavioral mechanisms contributing to parasite transmission to the next stage, such as movement in the environment *vs.* social isolation.

## 3. Inter-host stage and transmission potential

Most parasites are not immediately transmitted to a new host. Instead, they may be carried over and developed in vector hosts (biotic environment) or sit and wait in soil, water, or another abiotic environment before infecting a new host. The parasite must survive this intermediate stage to continue its life cycle and be exposed to a new host. The inability to withstand this environmental intermediate stage or develop the infective stage will result in an impaired parasite transmission rate and success. The importance of survival is obvious for parasites with free-living stages and vector-borne parasites. Long-lived resting stages are slowly degraded outside the host, and vector-borne parasites must survive the insect immune response long enough to complete development and produce transmission stages. Survival in the outside environment is also critical for parasites that are directly transmitted. SARS-CoV-2 viruses, for example, are transmitted in droplets and survive for only a short amount of time [77–79].

The intermediate transmission stage outside the primary host can significantly impact the parasite life [80] cycle and transmission potential (*T*_*p*_). We defined *T*_*P*_ as the number of infective cells that will have the opportunity to infect a new host, if it gets in contact with it. It, therefore, represents the subset of *T*_*A*_ that is able to survive the between-host environment. An important aspect of this framework is that the quality of the parasites at this stage (*Q*_*p*_) is heavily influenced by the environment in which they were produced and their adaptability to specific conditions. *Q*_*p*_ is affected by parasite taxa and the trade-offs associated with the parasite’s development in its initial host. For instance, lines of the parasite *Vavraia culicis* can have a negative correlation between parasite growth within the host and survival outside of the host [81]. Mortality at this stage is also influenced by the favorability of the environment (*Q*_*e*_). This environment can be anything outside the primary host: (i) a vector host, (ii) a water stream, or (iii) a surface. Nevertheless, using the same model as an example, *V. culicis*, which has a relatively long intermediate stage, is highly sensitive to abiotic factors such as temperature and UV light [82], which can significantly reduce its *T*_*p*_ [81]. Similarly, in vector-borne diseases, the mosquito’s nutrition can impact the development of malaria parasites within the vector [5]. Both factors can have aggravated costs/benefits with increased time in the environment (t) and, therefore, prolonged exposure to the factors. These factors can also be applied to vector-borne diseases if we think of them as generic descriptions of complex processes of vector-borne transmission. Thus, *Q*_*e*_ can refer to processes like the immune response of a vector or its mortality rate. *Q*_*p*_ is linked to the growth rate of the parasite in its vector, and *t* is the developmental time of the parasite in its vector. The two latter factors (*Q*_*p*_ and *t*) may also be linked to the first transmission stage within the host.

According to life-history theory [83,84], investment in one stage of a parasite’s life cycle often involves trade-offs that might affect subsequent stages. So, it is expected that a high parasite load within a primary host is linked to a reduced ability of the parasite to endure different environments. For instance, *Plasmodium* parasites produce more gametocytes, increasing their infectiousness to other mosquitoes [85], but this increase comes at the expense of reduced survival and longevity inside a vector [86]. A similar result is observed in a schistosome parasite whereby higher parasite growth in the final mammal host is associated with lower growth in the intermediate snail host [87].

The importance of such trade-offs is crystallized in the Curse of the Pharaoh hypothesis. The latter posits that infective cells that are able to live for a long time in the environment can exhibit high levels of virulence [88–90]. This hypothesis implies then that in some cases, the usual trade-off between virulence and transmission rate might be less pronounced, or they might be decoupled, challenging the traditional virulence trade-off theory. Furthermore, this hypothesis reinforces the influence of the intermediate between-host environment on the parasite’s transmission strategy. Although the Curse of the Pharaoh hypothesis remains relatively unexplored, a meta-analysis has identified examples of the nature of such phenomena [90]. This study also concluded that the relationship between virulence and environmental persistence is often taxa-specific [90] and likely driven by the unique evolutionary histories of each parasite. Nonetheless, this hypothesis suggests that we may be missing important aspects of the transmission process by not closely examining its stages and how they interact with parasitic traits [40,41]. Theoretical work indicates that additional factors, such as epidemiological dynamics and within-host competition among parasites, are vital for understanding virulence evolution [56,88,89]. Whether long-lived parasites evolve to be more or less virulent depends on the trade-off between virulence and longevity during their free-living stage [91,92] and the environment [93]. Distinguishing between classical transmission metrics and transmission potential can enhance our understanding of disease spread and virulence evolution. Here, we explicitly describe this intermediate stage of transmission among hosts and propose a simplified framework adaptable to most parasites:


$$T_{p}=T_{A}\left[1–µ\left(Q_{e},Q_{p},t\right)\right]$$

where *µ* is the parasite’s mortality during the inter-host stage, *Q*_*e*_ and *Q*_*p*_ indicate the quality of the environment and the parasite, respectively, and *t* is the time spent in this environment. The framework proposed here considers the impact of different ecological and evolutionary effectors on transmission potential.

**Fig 2:**
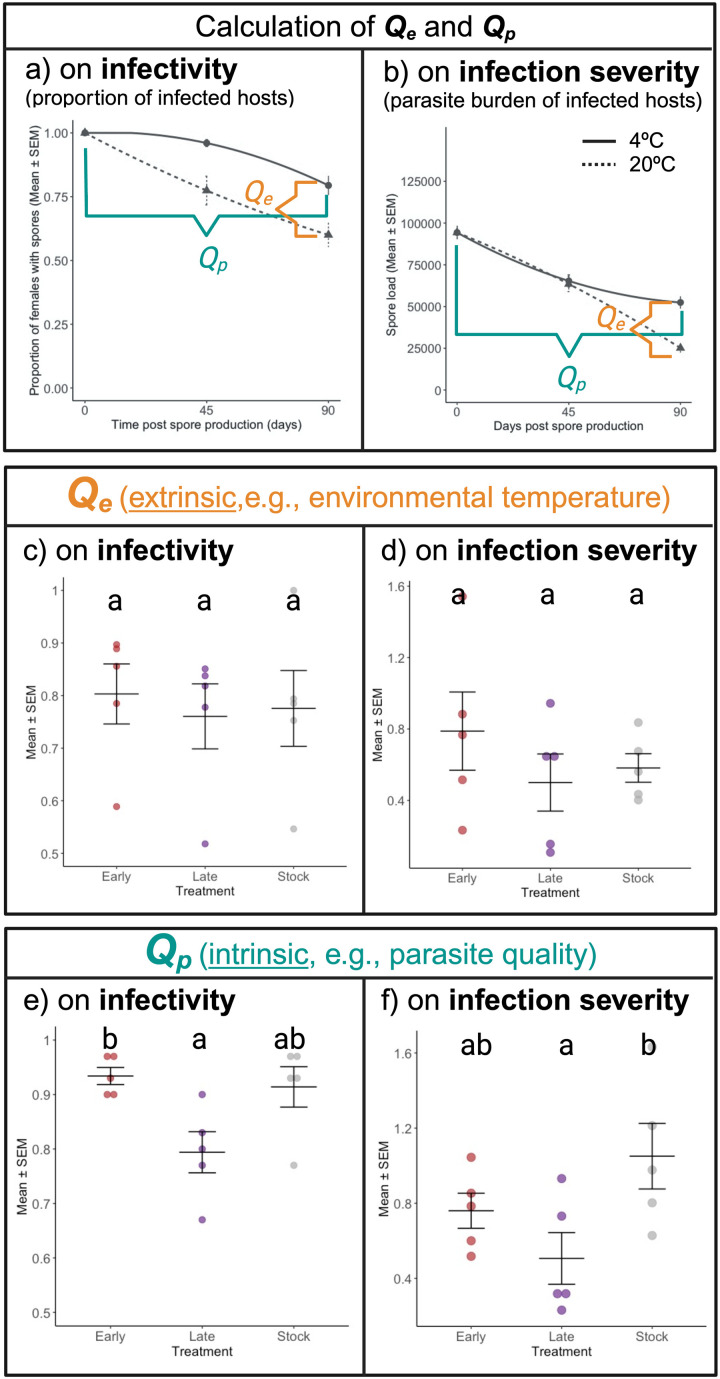
An empirical example of this framework. Silva and Koella (2024) selected the parasite *Vavraia culicis* for early or late transmission within the natural host *Anopheles gambiae*. Differently selected lines presented different levels of virulence within the host [94], which contrasted with different survival outside of the host [81]. To quantify the impact of the parasite quality (*Q*_*p*_) and environment (*Q*_*e*_), we used the framework presented in this article. The effects of *Q*_*p*_ and *Q*_*e*_ on (**a**) infectivity (proportion of infected individuals) and (**b**) infection severity (parasite burden of infected individuals) were calculated as demonstrated in the figures (**a**, **c**). *Q*_*p*_ was measured as the mean difference in the proportion of females with spores/spore load between infection with 90-days and 0-days old spores at 4 °C. *Q*_*e*_, as the effect of environment temperature in this model, was calculated by subtracting the mean proportion of females with spores/spore load after exposure to spores kept at 20 °C for 90 days from the mean proportion of females with spores/spore load at 4 °C for 90 days. The resulting means of each replicate from each treatment were then used in (**c**–**e**). The effects of parasite selection on the parasite spore quality (*Q*_*p*_) and environmental temperature (*Q*_*e*_) for (**c**, **e**) infectivity and (**d**, **f**) infection severity (i.e., spore load). Each data point represents the mean value of one of the five replicated selection lines. Letters denote significant differences between treatments according to the multiple comparisons test. Further information can be found in [81].

Here, we provide an example of the applicability of this framework, as conducted by Silva and Koella (2024) (Fig 2). In brief, the parasite *V. culicis* was selected for early- or late-transmission within the host *Anopheles gambiae*, or not (i.e., stock treatment) [94]. The differently selected parasite lines resulted in different levels of virulence within the host [94], with late-selected followed by early-selected and then stock. Hence, we applied this framework to measure their survival and which intrinsic (*Q*_*p*_) and extrinsic (*Q*_*e*_) factors impact their survival in the environment outside of the host throughout 90 days and at one of two temperatures, i.e., 4 °C and 20 °C. The effect of *Q*_*p*_ and *Q*_*e*_ on both infectivity (i.e., the proportion of secondarily infected hosts) and infection severity (i.e., parasite burden for those infected) were calculated as demonstrated in Fig 2a and 2b. Regarding infectivity, while *Q*_*p*_ was estimated by subtracting the number of successful transmissions on day 90 by the respective value for 0 days, *Q*_*e*_ was calculated as the difference in successful transmissions on day 90 between 20 °C and 4 °C. The same was performed for infection severity, but instead of the number of successful transmission events, the parasite burden of infected hosts was used. Through the use of this framework, we were able to explain parasite differences in survival outside of the host and, more importantly, that the differences are intrinsic to the parasite, meaning in spore quality and not due to environmental conditions [81].

## 4. Susceptibility of new host and transmission success

The last transmission stage covers parasites that survived the intermediate stage between hosts and therefore might be exposed to a new primary host, and potentially successfully infect it. If we call the probability of infecting the next host *β*_*p*_′, overall transmission (thus, *V*) becomes:


$$V=T_{p}\;\times \beta_{p}’$$

or


$$V=T_{A}\left[1–µ\left(Q_{e},Q_{p},t\right)\right]\times \beta_{p}’$$

and ergo:


$$V=\beta_{p}\times \beta_{c}\times I_{P}\left[1–µ\left(Q_{e},Q_{p},t\right)\right]\times \beta_{p}’$$

Note that *β*_*p*_′ depends on the susceptibility of the new host [15], which can be on factors such as life history [95,96], the immune strategy employed [53,54], the host’s genotype [15,97,98], and overall parasite fitness. *β*_*p*_′ can also depend on the quality of the parasites (*Q*_*p*_), which depends on the previous two stages and is affected by, for example, the first host’s nutrition, genotype and immune response [5,94,99,100] and the between-host environment [101,102]. Finally, *β*_*p*_′ can depend (non-linearly) on the number of parasites in the intermediate stage.

## 5. Concluding remarks and future directions

Transmission is a critical process of infection. Transmission rate influences parasite and host fitness in the short- and long-term, as well as at an individual and populational level. All these factors can determine the spread of disease and the rate and direction of evolution. Recent work on decomposing [40,103] and extensively studying the components of infection [46,104] and their relationships [94,105,106] is crucial. We propose that incorporating the parasite’s life history across different stages of the transmission process, rather than relying solely on classical transmission rate metrics, could improve predictions of infection outcomes in new hosts. The framework developed here is simple and broadly applicable to various parasites and transmission types. While several factors, such as parasite dispersal [107,108], host social aggregation [109,110], and multiple biotic environments (e.g., various vector hosts), are often case-specific, they can be integrated into this framework during the intermediate between-host stage.

The insights and solutions discussed here have significant implications for epidemiology, zoonotic disease emergence, outbreak management, and for understanding virulence evolution. For instance, many “so-called” emerging diseases already have been circulating within human populations but remain below transmission levels high enough to be classified as emergent. While genetic tools alert us of the chances of a zoonotic jump, we do not have much information on which host and parasite factors contribute to an alarming increase in transmission rates. Without the latter, we are unable to fully avoid zoonotic jumps or transmission evolution in a susceptible population.

Although infection biology is entering a new era, a significant gap remains in understanding how host and parasite biology interact to drive heterogeneity in transmission. Our framework directly addresses this gap by allowing transmission to be dissected step-wise and then integrated as a whole. This approach has important implications for disease treatment (medicine), prevention, and prediction (epidemiology). As we move toward increasingly threatened by multi-resistant microbes, it is crucial to exercise greater caution as a species and consider investing in novel disease control strategies, such has been the case of host disease tolerance. However, while much has been hypothesized about the evolutionary implications of host tolerance, relatively little attention has been given to its impact on parasite evolution—particularly in scenarios where evolution favors higher transmission rates, such as superspreading or supershedding.

We cannot overstate the importance of virulence evolution theory and its far-reaching impact on fields essential to human society and nature survival. The ongoing debate over the optimal theory of virulence and transmission evolution is unlikely to be settled soon, given the vast diversity of parasite infection strategies and life cycles—many of which remain poorly understood or entirely unknown. Through the dissection of different components of the transmission process, particularly transmission potential, we may uncover further evidence supporting the trade-off proposed by Anderson and May [2]—or we may not. Ultimately, the dynamics and constraints of infection play a crucial role in shaping transmission. Equally important is identifying which trade-offs could help pinpoint the most effective stages of transmission to target when designing control strategies (and which factors increase or reduce it).

Nonetheless, we can strive for a framework that enables the comparison of diverse parasite taxa under a unified model, which allows transmission to be analyzed as a whole or by individual transmission stages. The framework proposed in this article aims to achieve this while also establishing a standardized cross-disciplinary terminology applicable across various infections and parasite life cycles. Beyond advancing our understanding of infection and parasite evolution, this approach hopes to invite researchers from different fields to critically assess the limitations of current study models and explore new directions for future research in the field.
